# A Starch-Milk Paste Enables the Incorporation of Ripened Cheese in Novel Fresh Cheese

**DOI:** 10.17113/ftb.59.04.21.7262

**Published:** 2021-12

**Authors:** Elpiniki Palyvou-Gianna, Tatiana Paula Vilela, Ana Maria Gomes, João Paulo Ferreira

**Affiliations:** 1 Universidade Católica Portuguesa, CBQF - Centro de Biotecnologia e Química Fina – Laboratório Associado, Escola Superior de Biotecnologia, Rua Diogo Botelho 1327, 4169-005 Porto, Portugal; 2 Present address: Mondelez International, Research, Development & Quality, Birmingham, UK

**Keywords:** novel fresh cheese, cheese surplus valorisation, physicochemical analysis, sensory analysis

## Abstract

**Research background:**

Fresh cheese varieties represent an important share of the whole cheese market. Although with great variability in terms of composition and method of preparation, fresh cheese varieties are bland in flavour and their production originates whey drainage. On the other hand, the cheese market is also responsible for a significant amount of food waste. These motivated the development of a novel fresh cheese incorporating ripened cheese, which can then represent a valorisation of ripened cheese surpluses.

**Experimental approach:**

A variable amount of ripened cheese was dispersed in a paste of gelatinized starch (normal corn or waxy rice) in milk, producing melted cheese bases. These cheese bases were diluted with milk, sometimes enriched with skimmed milk powder, and then renneted. Macronutrient content and physical properties of the resultant fresh cheese were characterized. Sensory analyses of samples incorporating mature Cheddar, goat’s or ewe’s cheese were carried out.

**Results and conclusions:**

Gel formation of the initial mixture was hindered above 8% (*m*/*m*) incorporation of ripened cheese, which could be overcome by the addition of skimmed milk powder. These observations are corroborated by the hardness values from texture analysis tests. Evaluation of syneresis of different samples enabled to conclude that the addition of 2% (*m*/*m*) starch and 2.8% (*m*/*m*) skimmed milk powder contributes to reduction of its magnitude by half. Sensory analysis with a consumer panel indicated a preference for a more consistent texture of the fresh cheese, and for the Cheddar flavour.

**Novelty and scientific contribution:**

A novel fresh cheese variety incorporating dispersed ripened cheese was prepared. The proposed method is versatile and quite straightforward and does not use polyphosphate salts or originate whey wastage. The fresh cheese physical and sensorial properties can be manipulated by the amounts and types of added starch, ripened cheese and skimmed milk powder; such tailoring of fresh cheese properties widens product portfolio capacity for a larger number of consumer groups. The added ripened cheese can come from non-sellable pieces and unsold stocks from the retail sector, contributing to a reduction of food waste.

## INTRODUCTION

According to FAO, food waste is a global issue with a significant carbon footprint - as high as 3.3 billion tonnes of carbon dioxide per year released to the atmosphere. Moreover, the global volume of food waste is estimated at 1.6 billion tonnes of primary product equivalents (*1*). In recent years, waste valorisation coming from the food chain has gained much attention. In 2015, the European Commission presented ways to enhance the transition to a circular economy through recycling and reuse (*2*), and most recently adopted the new Circular Economy Action Plan (2020), Europe’s new agenda for sustainable growth, which introduces different measures fostering sustainable consumption. Cheese consumption in the European Union (and other parts of the World) has been increasing, with a yearly consumption per capita in 2019 close to 15 kg (*3*). Of this, a significant share comes from different varieties of fresh cheese (*e.g*. fromage blanc, crème fraîche, cottage cheese, quark, queso fresco, *etc*.) and from spreadable processed cheese. These products are appreciated for a variety of factors, such as freshness, lightness, versatility to be mixed with a variety of other foods or even beverages, *etc*. As they do not undergo a ripening stage, their flavour is generally bland.

The production process of fresh cheese is relatively simple and fast: after pasteurization and cooling, the milk, or milk-cream mixture, is standardized, and the coagulation takes place. The coagulation can be acid- and/or rennet-induced. Along with the coagulating factors, other ingredients, such as preservatives and emulsifiers, can be introduced (*4*). In order to remove the whey from the curd, draining or centrifugation of the coagulum can be carried out. To avoid the removal of whey at this stage, a more recent technology can be applied, namely ultrafiltration of the starting milk, which separates the smaller lactose, water, mineral and vitamin molecules from the larger proteins and fat globules. This results in a milk product concentrated in protein and lower in lactose content (*5*, *6*), but this process also generates a stream of nutritionally valuable liquid (*7*).

The current research study aims at the development of a novel renneted fresh cheese product incorporating (ripened) cheese. The latter may come from the surpluses of the dairy industry and/or the retail sector, thus representing a needed mitigation of food waste, since food waste in the dairy sector in Europe can be over 10%, and in North America and Oceania over 20% (*8*). The ripened cheese can provide additional nutritional value to the fresh cheese, particularly added protein, lipids and minerals. It will also bring a multitude of flavour and aroma compounds, as well as varying textures.

Ripened cheese can be dispersed into submillimetre particles when mixed in a hot paste of gelatinized starch, being herein designated melted cheese base. Such dispersions are used, with variants, in the preparation of some simple cheese sauces (*9*-*11*). This technique for dispersing matured cheese is an interesting alternative to the use of emulsifying salts, namely polyphosphates, generally employed in processed cheese (*10*, *11*). Polyphosphates can be deleterious to health, at least for certain groups, such as patients with bone disease (*12*), besides imparting a metallic tone to the processed cheese (*13*).

Furthermore, the process proposed here dispenses the step of cutting the coagulum and removal of whey, which can be time consuming and costly, and that produces abundant whey wastage. In this process, the milk whey is incorporated in the final product, contributing with minerals and proteins of high biological value.

Therefore, the aim of this study is the development and testing of a new fresh cheese incorporating ripened cheese, dispersed in a paste of gelatinized starch. The nature of the ripened cheese and the type of starch (corn or waxy rice starch) were studied.

## MATERIALS AND METHODS

### Materials

For the sample preparation, the milk used was semi-skimmed (1.5% (*m*/*m*) fat) high-temperature short-time (HTST) pasteurised milk (Vigor, Porto, Portugal). The starches employed were regular corn (Maizena, Unilever, Lisboa, Portugal) and waxy rice (Remyline XS, BENEO GmbH, Mannheim, Germany, kindly provided by Nutripar, Porto, Portugal). The types of cheese were Emmental (Milbona, Bissingen, Germany), goat’s (Queso de Cabra, Lácteas García Baquero S.A., Alcázar de San Juan, Ciudad Real, Spain), ewe’s (Queijo Serra Valmadeiros, Indulac Industrias Lácteas S.A., Oliveira de Azeméis, Portugal) and mature Cheddar (Valley Spire, Dale Farm Ltd., Belfast, UK). Skimmed milk powder was from Regilait (Nestlé S.A, Vevey, Switzerland). The rennet was a liquid preparation of *Rhizomucor miehei* enzyme (Britex, Lusocoalho, Montes da Senhora, Portugal).

### Preparation of melted cheese base

For a typical preparation, corn starch was dispersed in cold milk, at a ratio of 5 g per 100 g milk, in a small non-adherent pan, and the mixture was heated on a hotplate for approx. 5 min, with continuous hand stirring, until reaching 85 °C. Temperature was controlled with a kitchen electronic thermometer (LACOR, Bergara, Spain). At this point, the gelatinization of the starch was noticeable and 20 g of grated or finely cut cheese were added to the milk-starch slurry. The mixture was removed from the hotplate and hand stirred for approx. 30 s, until the cheese was fully dispersed, with no visible macroscopic pieces. This melted cheese base was left to cool down to room temperature (approx. 21 °C).

When using waxy rice starch, the procedure was similar, but the milk-starch mixture was heated to 90 °C and kept for 2 min in order to achieve complete starch gelatinization before cheese addition.

### Light and fluorescence optical microscopy

For analysing the microstructure of the melted cheese bases prepared with grated Emmental cheese and starch, an Olympus BX51 (Olympus, Tokyo, Japan) optical microscope was used, with a 10× objective lens, and the images were captured using a Power Shot G10 digital camera (Canon, Tokyo, Japan). In order to visualize starch and protein, samples were stained with Rhodamine B (1 g/L) (*14*, *15*), and to visualize fat, with Sudan III (1 g/L) (*16*). Afterwards, aliquots were transferred to concave slides and covered with a cover slip. Samples were equilibrated at room temperature for 15 min prior to microscopic analysis, using the fluorescence mode with Rhodamine B and the normal light mode with Sudan III samples. All observations were performed in duplicate, with at least four pictures taken per sample. Software ImageJ (*17*) was used to evaluate the sizes of the protein agglomerates.

### Sample preparation

Four different control mixtures (without cheese) using milk and the two types of starch were prepared, namely, just HTST milk (sample CT), milk with 2% (*m*/*m*) corn starch (CT.CS.1) or 2% (*m*/*m*) waxy rice starch (CT.WRS.1), milk with 4% (*m*/*m*) starch (CT.CS.2 and CT.WRS.2), and milk with 2% (*m*/*m*) starch and 2.8% (*m*/*m*) skimmed milk powder (CT.CS.3 and CT.WRS.3). In order to prepare these samples, milk, milk with 4% (*m*/*m*) gelatinized starch, and milk with 5.7% (*m*/*m*) skimmed milk powder were used and mixed in different proportions, according to Table 1. The last solution was prepared by adding 6 g skimmed milk powder to 100 g fluid milk, and left for 24 h at 4 °C for the powder to fully rehydrate.

**Table 1:** Mixing of different preparations in the formulation of control and fresh cheese samples

Sample	*m*(milk)/g	*m*(milk with 4% starch)/g	*m*(milk with 5.7% SMP)/g	*m*(MCB with 16% cheese)/g
CT	100	0	0	0
CT.CS.1 CT.WRS.1	50	50	0	0
CT.CS.2 CT.WRS.2	0	100	0	0
CT.CS.3 CT.WRS.3	0	50	50	0
EM.CS.1 EM.WRS.1	50	30	0	20
EM.CS.2 EM.WRS.2	50	20	0	30
EM.CS.3 EM.WRS.3	50	10	0	40
EM.CS.4 EM.WRS.4	50	0	0	50
EM.CS.5 EM.WRS.5	0	0	50	50
EW.CS.1 EW.WRS.1	50	30	0	20
EW.CS.2 EW.WRS.2	50	20	0	30
EW.CS.3 EW.WRS.3	50	10	0	40
EW.CS.4 EW.WRS.4	50	0	0	50
EW.CS.5 EW.WRS.5	0	0	50	50

MCB=melted cheese bases, CT=control milk, CT.CS and CT.WRS=control milk with corn starch (CS) or waxy rice starch (WRS), EM.CS.1 to 5 and EM.WRS.1 to 5=fresh cheese samples with varying amounts of Emmental cheese (EM) and CS or WRS, EW.CS.1 to 5 and EW.WRS.1 to 5=fresh cheese samples with varying amounts of ewe’s cheese (EW). Samples CT.CS.3, CT.WRS.3, EM.CS.5, EM.WRS.5, EW.CS.5 and EW.WRS.5 also contain skimmed milk powder (SMP)

For samples containing ripened cheese, we further used a melted cheese base incorporating 4% (*m*/*m*) corn starch and 16% (*m*/*m*) Emmental cheese. Then, samples with an amount of cheese varying between 3.2 and 8.0% (*m*/*m*) (samples EM.CS.1 to 5) were prepared (Table 1). One of the samples (EM.CS.5) included also skimmed milk powder at 2.8% (*m*/*m*). Similar samples were prepared with waxy rice starch (EM.WRS.1 to 5) (Table 1). Two other sets of samples were prepared with ewe’s cheese, using either corn starch (EW.CS.1 to 5) or waxy rice starch (samples EW.WRS.1 to 5) (Table 1).

The renneting of all samples was done by adding 240 μL liquid rennet per 100 g of mixture, pre-equilibrated in a thermostatic water bath (TW20; JULABO GmbH, Seelbach, Germany). The temperature was maintained at 35 °C for 15 min (until coagulation was completed). The flasks with the samples were then cooled down to 4 °C. Several batches of all samples were prepared for the following tests.

### Determination of total solids

The determination of the total solids was carried out for a representative group of samples, namely for the ewe’s and Emmental ripened cheese, and for fresh cheese samples EW.CS.4 and EM.CS.4. Aluminium dishes were pre-dried (105 °C, 1 h) and weighed. A mass of 3-5 g of sample was placed in the dishes and the mass was recorded. The samples were dried in an oven to constant mass (drying oven d-6450; Heraeus Thermo Fisher Scientific, Waltham, MA, USA) for 24 h at 105 °C. The samples were cooled in the desiccator and the total solids were determined according to AOAC method 925.23 (*18*), using the following equation:



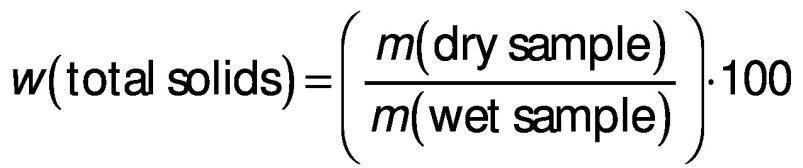



Two batches of each sample type were subjected to analysis and, in each case, duplicate measurements were taken. Results are expressed as mean value±S.D.

### Determination of protein content

The total protein content in the Emmental and ewe’s cheese was determined in duplicate samples using a non-conventional method. It is based on the one proposed by Reichardt and Eckert (*19*), but with modifications. A standard casein solution was prepared by adding 0.405 g of bovine casein (Sigma-Aldrich, Merck, St. Louis, MO, USA) in 50 mL of deionized water, followed by heating to 40 °C and gently stirring. The absorbance at 280 nm of this solution and several dilutions was determined (Spectronic Helios Gamma UV-Vis spectrophotometer, Thermo Fisher Scientific) and a calibration curve of protein concentration was thus obtained.

For the determination of the protein content of cheese, about 1.33 g of cheese cut in small pieces was placed in 30 mL of 0.1 M NaOH and left overnight. The following day, the mixture was placed in a water bath (TW20; JULABO GmbH) at 40 °C for 10 min and mixed well. After cooling down, it was centrifuged (centrifuge Universal 320R; Andreas Hettich GmbH, Germany) at 4000×*g* and 4 °C for 10 min. The top layer of fat was removed, the underlying supernatant was collected and its volume evaluated. The absorbance at 280 nm was measured using Spectronic Helios Gamma UV-Vis spectrophotometer (Thermo Fisher Scientific), by diluting 80 μL of the supernatant with 920 μL of 0.1 M NaOH. The concentration was calculated from the casein curve, and then the protein content in cheese calculated. The determinations were done in duplicate samples of cheese and the mean values±S.D. were calculated. We have shown that this method gives results that are not statistically different from those obtained with the standard Kjeldahl method (*20*).

### Determination of fat content of ripened cheese

The fat content of the different types of ripened cheese was measured by the Van Gulik method (*21*). The cheese was grated or cut in small pieces, and precisely 3 g were placed in the lower part of the Van Gulik butyrometer (cheese butyrometer 0-40%; Gerber Instruments, Effretikon, Switzerland). A volume of 15 mL of 62% sulfuric acid (*ρ*=(1522±0.005) g/mL), according to Van Gulik, was added, the butyrometer was tapped and put in a water bath at 65 °C until the cheese was fully digested. Then, 1 mL of isoamyl alcohol was added and the contents were mixed thoroughly. Additional sulfuric acid was added, up to the point that the liquid reached the 35% mark of the butyrometer scale. The butyrometer was tapped and the contents were mixed thoroughly once again. The samples were centrifuged in a Gerber centrifuge (NormMilk centrifuge; International PVI, Milan, Italy) for 6 min at 1200 rpm and 65 °C. After the centrifugation, the fat content was read directly on the butyrometer. The determinations were done in duplicate and the mean values±S.D. were calculated.

### Determination of syneresis

Control samples and fresh cheese samples EM.CS.2, EM.CS.4, EW.WRS.2 and EW.WRS.4 were centrifuged (centrifuge Universal 320R; Andreas Hettich GmbH) at 1500×*g* for 15 min at 20 °C (*22*), and the mass of the pellet and supernatant (separated whey) was measured. The syneresis was calculated as follows (*23*):



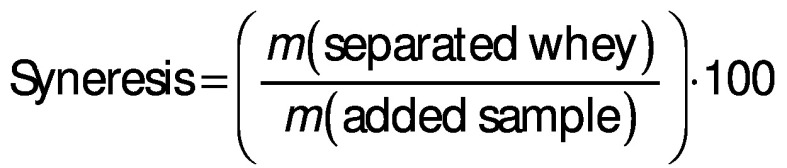



Two batches of each sample type were subjected to analysis and, in each case, duplicate measurements were taken. Results are expressed as mean value±S.D.

### Texture profile analysis

For the texture profile analysis, the renneted samples rested at 4 °C for 24 h, and then they were equilibrated at 15 °C. A Stable Micro Systems TA.XT Plus Texture Analyzer (Surrey, UK) with a cylindrical probe of 36 mm diameter was used. The fresh cheese samples were tested in the glass flasks of approx. 6 cm diameter in which they were renneted, without any transfer to avoid gel breakage. A typical texture profile analysis cycle was used, with a trigger force of 5 g (corresponding to 0.049 N). The probe test speed was 1 mm/s and the maximum deformation was set at 12 mm, which corresponded to 33-40% of sample height. The waiting time between the two compressions was 5 s (*24*, *25*). The measurements were done in samples of three distinct batches, as this is a destructive test. The mean value±S.D. of each sample type was calculated.

### Sensory analysis

The sensory analysis was carried out by a consumer panel of 40 people from the University community, aged between 15 and 45. Fresh cheese (600 mL each, in 1-litre glass flasks) containing 6.4% of four different (ripened) types of cheese was prepared: goat’s cheese (sensorial sample S.GT), ewe’s (S.EW) and two with Cheddar cheese, with and without skimmed milk powder at 2.8% (sensorial samples S.CH.SMP and S.CH). The starch selected for this purpose was the waxy rice, as preliminary trials showed that it led to fresh cheese with a smoother texture and no perceptible starch taste. Also, although the physicochemical studies reported here used Emmental cheese, the sensorial tests employed the stronger flavoured Cheddar, and included a goat’s cheese. After resting overnight at 4 °C, the fresh cheese samples were equilibrated in a water bath (TW20; JULABO GmbH) at 8 °C and maintained at this temperature throughout the session. The panellists were served with a spoonful of each sample in a small plastic cup, upon arrival. The samples were identified by randomized codes. The panellists were asked to give ratings on a 9-point hedonic scale for each product, regarding the following attributes: appearance, odour, texture, flavour and overall evaluation of the product. They were asked in addition to evaluate the intensity of the flavour of each sample as too weak, ideal or too strong.

### Statistical analysis

The statistical analysis of these results was made using a one-way ANOVA test, with the application of the Tukey’s test for pairwise comparisons between particular samples. The normality of the data, as well as the homogeneity of variances was verified, and the SPSS software (*26*) was used for the statistical analysis of the results.

## RESULTS AND DISCUSSION

### Microstructure of melted cheese base

Ripened cheese can be dispersed into sub-millimetre particles by mixing it in a hot paste of gelatinized starch in milk. This melted cheese base has been under study in our laboratory, in particular its applications. It can be an ingredient for the development of different food products incorporating ripened cheese, which will provide added nutritional value, texture and flavour to the final product, while preventing potential food waste. Fig. 1 shows microscopic images of melted Emmental cheese bases prepared with corn starch (Figs. 1a and 1c) and waxy rice starch (Figs. 1b and 1d).

We used light microscopy, together with particular dyes, to analyse the microstructure of melted Emmental cheese bases. The dye Rhodamine B (Figs. 1a and 1b) stains proteins orange and starch yellow (*14*). We can see clearly starch layers surrounding dispersed cheese protein aggregates. Furthermore, some remnants of starch granules are also visible. The protein aggregates have a broad size range, with a larger length varying from 70 to 900 μm. The surrounding starch points to a strong interaction with the cheese protein. The fact that the cheese protein matrix is not completely dissociated might be due to the protein matrix breaking at the weaker points of interaction among strands, or to the protein matrix breaking at those points susceptible to stronger interactions with the gelatinized starch.

The dye Sudan III (Figs. 1c and 1d) stains fat in red. We see that the fat forms globules entrapped in the cheese matrix (*27*), with sizes ranging from ~1 up to 70 μm in corn starch samples, and up to 50 μm in waxy rice starch samples, which is in accordance with reported fat globule sizes in cheese (*28*, *29*). Furthermore, the fat globules are spherical in shape. Same zones show a diffuse staining from dispersed fat in the matrix, resultant from the high temperatures and the shearing employed in the preparation of melted cheese bases (*30*).

We have recently demonstrated (*20*) that the matrix of this same Emmental cheese is primarily held by a combination of hydrophobic and electrostatic interactions, including hydrogen bonds. Starch can readily form strong intermolecular and intramolecular hydrogen bonds, but it also has a hydrophobic character, provided by the segments of double helices formed by glucose chains joined together by glycosidic α-1,4 bonds (*31*-*34*). Therefore, we can speculate that the interactions between starch and the protein aggregates can also be dominated by electrostatic and hydrophobic bonds (*31*, *35*). These mutual interactions are the foundation for the cheese-dispersing capability of gelatinized starch. Future studies are needed to study specifically this feature in more depth.

### Key variables in fresh cheese preparation

**Fig. S1:**
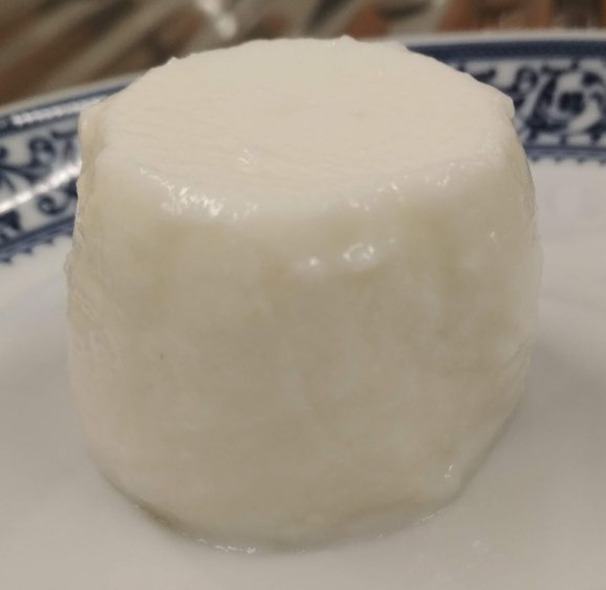
Image of a fresh cheese sample containing Emmental cheese and skimmed milk powder (sample EM.CS.5)

In this research, a novel renneted fresh cheese was developed based on melted cheese bases. Supplementary material provides a photo of one such sample (Fig. S1). Fresh cheese is generally produced by renneting milk, or a mixture of milk and cream, and then draining the curd, in order to obtain higher contents of protein and fat. Alternatively, a few dairy industries start by concentrating the milk by ultrafiltration before renneting. In this latter case, the coagulation can be carried out already in the final package, and, in order to achieve a longer shelf life, the product can be further subjected to heat treatment. In either process, although the product has an increased protein (and fat) content, the flavour is bland compared to ripened cheese. Our technique enables the inclusion of ripened cheese - which could eventually become food waste - into the matrix of the novel fresh cheese, providing additional high value protein, fat, mineral salts, texture and flavour to the product. The production and consumption of fresh cheese varieties have been facing a steady and continuous increase over the past years and constitute a major proportion of the cheese consumed in many countries, including Portugal, where the traditional form of fresh cheese is designated queijo fresco. Fresh cheese varieties may vary from a softer creamy texture to a more continuous gel suitable for cutting. Extreme softness, brittleness or graininess, and lower yields are some of the drawbacks pointed out in conventional fresh cheese manufacture. Our technique enables the achievement of a more consistent and continuous curd at higher yields. It should also be noted that the quality and nutritional balance of fresh dairy products, including fresh cheese varieties, the versatility of their consumption (home cooking and processing use) and longer shelf-life strategies have been pointed out as important features to contribute to increasing EU net exports of fresh dairy products by 2030 (*36*). The novel fresh cheese developed herein contributes to this product competitiveness, where an improved nutritional profile (corresponding to the evolving consumer demands), a higher and much appreciated sensorial quality, an improved versatility of consumption, and a cost-effective production adaptability leading to the reduction of waste and higher yields are all contributing features, and in the medium-to-long term they lead to higher *per capita* consumption.

From a technological point of view, differences were observed in the production of conventional and melted cheese base-related renneted fresh cheese; the addition of fungal rennet to plain semi-skimmed milk resulted, after 20 min, in a soft gel that, when cut and slightly squeezed, surfaced whey, whereas the renneting of preparations with melted cheese bases was completed in approx. 15 min, a shorter time relative to the above control. No surfacing of whey was observed when these gels were cut.

Preliminary trials were carried out varying the amount of cheese and that of starch in the final mixture. Incorporation of cheese above a final mass fraction of approx. 8% resulted in a too soft, almost fluid texture of the fresh cheese. The conclusion is that the incorporation of dispersed ripened cheese inhibited gel formation. In fact, in the melted cheese base, the sub-millimetre cheese particles are coated with starch, as revealed by microscopy. It is thus understandable that these particles act as inert fillers, hindering gel formation by the renneted milk caseins. Therefore, the microstructure of the fresh cheese is envisaged as a typical fresh rennet gel (*14*, *37*) that incorporates dissolved starch fragments and dispersed starch-coated ripened cheese particles.

Having an amount of starch above 2% in the final mixture, although leading to stronger gels, can also lead to a perceptible starch taste in the product, particularly when corn starch is used. Therefore, based on these observations, standard fresh cheese samples had a fixed final starch mass fraction of 2% and an 8% limit for ripened cheese incorporation.

### Total solids in ripened cheese and in fresh cheese samples

The determination of dry matter content (total solids) was carried out for two types of ripened cheese and two representative fresh cheese samples incorporating 6.4% (*m*/*m*) of those same cheese varieties. These samples were selected for having identical content of ripened cheese as the ones in sensorial analysis. Duplicate measurements were carried out and the mean values±S.D. are presented in Table 2. As the total solids of the Emmental and ewe’s cheese were similar, the two fresh renneted types of cheese also had identical results, as expected. One-way ANOVA test was applied between the pairs of samples Emmental/ewe’s cheese and EM.CS.4/EW.CS.4, showing that the samples of each pair were not significantly different (p<0.05) in this regard. Regarding the fresh cheese samples, we note that the level of total solids is within typical values of several varieties of conventional fresh cheese, such as cottage cheese, ymer or fromage blanc (*38*, *39*).

**Table 2:** Dry matter mass fractions of two ripened cheese and two fresh cheese samples

Sample	*w*(total solid)
Emmental cheese	(61.6±0.6)^a^
ewe’s cheese	(57.8±0.2)^a^
EM.CS.4	(15.10±0.02)^b^
EW.CS.4	(15.1±0.2)^b^

Results are expressed as mean value±S.D. (*N*=4). Samples with the same superscripted letter are not statistically significantly different according to the Tukey’s test. EM.CS.4 and EW.CS.4=fresh cheese samples containing either Emmental cheese (EM) or ewe’s cheese (EW) and corn starch (CS)

### Total protein content in ripened cheese

Using our modified method of Reichardt and Eckert (*19*) with cheese dispersed in sodium hydroxide solution, followed by evaluation of the protein in the fat-free solution by UV absorbance, the protein content of Emmental cheese was (29.8±0.2) % and that of ewe’s cheese was (21.3±0.4) %. These results are very close to the values indicated on the product labels, namely 28 and 23%, respectively. This shows that the employed method is quite precise, and it can be used as an alternative to the lengthy Kjeldahl one. In fact, previous work in our laboratory has shown a good agreement between the two methods (*20*).

### Total fat in ripened cheese

The Van Gulik method applied to Emmental, ewe’s and goat’s ripened cheese indicated fat content of (29.00±0.07), (27.0±0.7) and (35.0±0.0) %, respectively. These values are also close to the ones indicated on the product labels (28, 30 and 35%, respectively).

The Van Gulik method was also carried out for samples of the fresh renneted cheese samples. However, probably due to interference of the starch, the method was not successful.

### Macronutrient composition of fresh cheese samples

Table 3 presents the macronutrient composition of fresh cheese samples incorporating Emmental and ewe’s cheese. The results were determined based on the corresponding compositions of the raw materials - milk, cheese and starches. We considered that the validation of the macronutrient composition of the starting materials – particularly the ripened cheese, as described above, is sufficient for a reliable estimate of the corresponding composition of the fresh renneted cheese. These have a more complex matrix, with some analytical methods, in particular fat determination, being more prone to interferences.

**Table 3:** Macronutrient composition of the fresh cheese samples

Sample	*w*(protein)/(g/100 g)	*w*(lipid)/(g/100 g)	*w*(carbohydrate)/(g/100 g)
EM.CS.1	4.2	2.4	6.9
EM.CS.2	4.6	2.9	6.9
EM.CS.3	5.0	3.3	6.9
EM.CS.4	5.4	3.8	6.6
EW.CS.1	3.9	2.4	6.9
EW.CS.2	4.2	2.8	6.9
EW.CS.3	4.5	3.2	6.9
EW.CS.4	4.8	3.6	6.8
EW.CS.5	5.4	3.6	7.8
EM.WRS.1	4.2	2.4	6.8
EM.WRS.2	4.6	2.9	6.8
EM.WRS.3	5.0	3.3	6.7
EM.WRS.4	5.4	3.8	6.7
EM.WRS.5	6.1	3.8	7.6
EW.WRS.1	3.9	2.4	6.8
EW.WRS.2	4.2	2.4	6.8
EW.WRS.3	4.5	2.8	6.7
EW.WRS.4	4.8	3.2	6.7
EW.WRS.5	5.4	3.6	7.6

EM.CS1 to 5 and EM.WRS1 to 5=fresh cheese samples with varying amounts of Emmental cheese (EM) and corn starch (CS) or waxy rice starch (WRS), EW.CS1 to 5 and EW.WRS1 to 5=fresh cheese samples with varying amounts of ewe’s cheese (EW). Samples with number 5 also contain skimmed milk powder (SMP)

The above-mentioned experimental results for total solids of samples EM.CS.4 and EW.CS.4 (15.1% for both) can be compared with the sum of macronutrients in Table 3, 15.8 and 15.2%, respectively. Although these last numbers do not incorporate the (minor) ash contents, the proximity of results supports our methodology.

The composition of this fresh renneted cheese can be compared to those of commercial products. A well-known commercial spreadable processed cheese (Philadelphia, Kraft Foods) has a protein content around 5.4% in the original version, and 7.4% in the light version (*40*). A review of different brands of fromage blanc on the French market points to an average protein content of 5.7% (*39*). In terms of fat content, the original Philadelphia has 21%, and the light version 11% (*40*), while the average of fromage blanc is 7.8% (*39*). Our samples have similar protein content, but the formulation can be easily adapted for higher protein content, by simply increasing the amount of ripened cheese and/or skimmed milk powder (see below). In terms of fat, our fresh cheese has significantly lower content than the Philadelphia (or other brands) cream cheese, and also lower than the standard fromage blanc, which can be envisaged as a health-promoting feature. These observations are also in agreement with a recent study of six commercial cream cheeses (*41*).

An issue can be raised regarding the salt content of the fresh cheese, as ripened cheese generally has a considerable amount. As the only ingredient contributing significantly to salt content is the ripened cheese, noting that all varieties used have salt mass fractions below 1.9 g/100 g, then it gives to the fresh cheese, at the amounts used, a salt mass fraction below 0.15 g/100 g, which is fairly low. We point out that there is no salting step in the preparation procedure.

The control samples of plain milk had a pH=6.4, and those of milk with added starch had pH=6.6. The samples with ripened cheese had, as expected, slightly lower pH, with values reaching 5.8 for those with the highest addition. These values are higher than those of common fresh cheese varieties, which, in most cases, are within 4.5–5.0 (*38*), as our samples did not undergo an acidification step, either by fermentation or acid addition.

### Level of syneresis

The determination was done for a meaningful set of samples, in duplicate, and the mean value±S.D. of the measurements is presented in Table 4. The control sample (CT), containing only renneted milk, shows the highest syneresis values. Incorporation of corn starch or waxy rice starch (CT.CS.1-3 and CT.WRS.1-3) leads to a decrease in syneresis, with the difference to the control being statistically significant (p<0.05) at the 4% mass fraction. Corn starch and waxy rice starch, at equal mass fractions, showed no significant differences between them (p>0.05), therefore, we can conclude that starch addition contributes to water retention in the gel and lower syneresis.

**Table 4:** Average values of syneresis of fresh cheese samples

Sample	*w*(starch)/%	*w*(ripened cheese)/%	*w*(SMP)/%	Syneresis/%
CT	-	-	-	(48.0±2.8)^a^
CT.CS.1	2	-	-	(23.0±0.1)^ac^
CT.CS.2	4	-	-	(11.0±2.1)^bc^
CT.CS.3	2	-	2.8	(21.0±3.6)^ac^
CT.WRS.1	2	-	-	(36.0±0.1)^ac^
CT.WRS.2	4	-	-	(10.0±0.3)^bc^
CT.WRS.3	2	-	2.8	(22.0±1.8)^ac^
EW.CS.2	2	4.8	-	(37.0±0.1)^ac^
EW.CS.4	2	8	-	(39.0±1.3)^ac^
EW.WRS.2	2	4.8	-	(42.0±0.0)^a^
EW.WRS.4	2	8	-	(40.0±0.2)^ac^

Results are expressed as mean value±S.D. (*N*=4). Samples with the same superscripted letter are not statistically significantly different according to the Tukey’s test. CT=control milk, CT.CS.1 and 2 and CT.WRS.1 and 2=control milk with corn starch (CS) or waxy rice starch (WRS), CT.CS.3 and CT.WRS.3 also contain skimmed milk powder (SMP). EW.CS.2 and 4 and EW.WRS.2 and 4=fresh cheese samples with ewe’s (EW) cheese

Addition of 2.8% (*m*/*m*) skimmed milk powder also seemed to contribute to an additional reduction in syneresis, when samples with the same amount of either corn starch or waxy rice starch (2%) are compared, although with no statistical significance (p>0.05). This observation deserves further studies using a higher amount of skimmed milk powder.

The addition of ripened cheese to fresh cheese samples prepared with corn starch leads to higher syneresis values (though not statistically significant), and in samples with waxy rice starch the addition of ripened cheese affected syneresis minimally, compared with corresponding controls. In both cases, increasing the cheese addition from 4.8 to 8% did not change syneresis significantly (p>0.05). Therefore, one can conclude that the presence of starch is the factor with the higher impact on syneresis, as seen also in previous studies (*42*, *43*).

### Textural profile of fresh cheese samples

Texture profile analyses were done in triplicate samples and the mean value±S.D. of the three measurements is presented in Table 5. The selected parameters were hardness, adhesiveness, springiness and cohesiveness.

**Table 5:** Results of textural analysis of fresh cheese samples

Sample	Hardness/ g	Adhesiveness/ (g·s)	Springiness	Cohesiveness
CT	(139.7±11.2)^acdf^	(-31.1±7.4)^a^	(0.97±0.01)^a^	(0.47±0.03)ª
CT.CS.1	(98.1±3.3)^acde^	(-63.6±3.2)^a^	(095±0.01)^a^	(0.51±0.00)^ab^
CT.CS.2	(166.5±12.3)^abf^	(-190.5±16.6)^bc^	(0.93±0.01)^b^	(0.50±0.01)^ab^
CT.CS.3	(146.8±16.9)^acf^	(-110.6±21.3)^a^	(0.95±0.00)^a^	(0.48±0.02)^ab^
CT.WRS.1	(104.9±15.8)^acde^	(-65.2±12.0)^a^	(0.95±0.02)^a^	(0.6±0.0)^c^
CT.WRS.2	(147.7±76.8)^acf^	(-259.0±151.9)^b^	(0.91±0.03)^b^	(0.50±0.00)^b^
CT.WRS.3	(227.3±27.6)^bf^	(-209.8±1.8)^bd^	(0.94±0.00)^ab^	(0.52±0.00)^a^
EM.CS.1	(94.0±7.0)^acde^	(-63.1±8.7)^ace^	(0.96±0.00)^ab^	(0.48±0.00)^ab^
EM.CS.2	(77.4±6.1)^cde^	(-50.9±11.8)^ace^	(0.96±0.00)^ab^	(0.51±0.00)^ab^
EM.CS.3	(67.6±0.4)^de^	(-43.2±3.6)^ac^	(0.96±0.00)^ab^	(0.53±0.00)^ab^
EM.CS.4	(54.7±1.4)^e^	(-26.7±1.5)^ac^	(0.97±0.00) ^ab^	(0.61±0.00)^ab^
EM.CS.5	(99.1±4.8)^acde^	(-69.8±12.0)^acd^	(0.95±0.00)^ab^	(0.52±0.00)^ab^
EW.CS.1	(84.4±3.0)^cde^	(-49.8±7.8)^ace^	(0.96±0.01)^ab^	(0.52±0.04)^ab^
EW.CS.2	(79.5±4.8)^cde^	(-54.4±3.0)^ace^	(0.96±0.00)^ab^	(0.51±0.03)^ab^
EW.CS.3	(63.2±8.8)^de^	(-31.5±11.2)^ac^	(0.97±0.00)^ab^	(0.56±0.01)^ab^
EW.CS.4	(57.3±3.3)^e^	(-24.6±9.3)^ac^	(0.97±0.01)^ab^	(0.58±0.03)^ab^
EW.CS.5	(118.5±2.0)^acdef^	(-84.6±12.3)^acd^	(0.97±0.02)^ab^	(0.46±0.01)^ab^
EW.WRS.1	(107.6±10.0)^acdef^	(-92.5±134.6)^acd^	(0.94±0.00)^ab^	(0.53±0.02)^ab^
EW.WRS.2	(97.6±6.5)^acde^	(-77.2±115.8)^acd^	(0.94±0.01)^ab^	(0.53±0.01)^ab^
EW.WRS.3	(85.7±4.5)^cde^	(-63.9±99.9)^ace^	(0.95±0.00)^ab^	(0.54±0.02)^ab^
EW.WRS.4	(72.8±3.6)^cde^	(-47.1±79.5)^ace^	(0.95±0.00)^ab^	(0.57±0.00)^abc^
EW.WRS.5	(183.0±2.8)^f^	(-184.9±31.3)^bde^	(0.94±0.00)^ab^	(0.47±0.00)^ab^

Results are expressed as mean value±S.D. (*N*=3). Samples with the same superscripted letter within the same column are not statistically significantly different according to the Tukey’s test. CT=control milk, CT.CS and CT.WRS=milk control with corn starch (CS) or waxy rice starch (WRS), EM.CS.1 to 5 and EM.WRS.1 to 5=fresh cheese samples with varying amounts of Emmental cheese (EM) and CS or WRS, EW.CS.1 to 5 and EW.WRS.1 to 5=fresh cheese samples with varying amounts of ewe’s cheese (EW). Samples CT.CS.3, CT.WRS.3, EM.CS.5, EM.WRS.5, EW.CS.5 and EW.WRS.5 also contain skimmed milk powder (SMP)

The force-deformation curves (not shown) revealed that all samples fractured near or at the hardness level (where the maximum force was applied), except sample CT.WRS.3, which did not fracture. The majority of the cheese samples fractured between 55 and 100 g (approx. 0.539 and 0.981 N) applied force. The fact that CT.WRS.3 did not fracture resides in the particular resistance of this gel, provided by both the gelatinized waxy starch and the additional rennetable casein from skimmed milk powder.

Regarding hardness, the addition of 2% starch to control samples (CT.CS.1 or CT.WRS.1) led to slightly lower values than those of the control milk (CT), but 4% starch (CT.CS.2 or CT.WRS.2) reverted the trend; however, the differences are not statistically significant (p>0.05).

In control samples with added skimmed milk powder (CT.CS.3 and CT.WRS.3), the one prepared with waxy rice starch showed a significant difference (p<0.05) from the other control samples. As said above, skimmed milk powder increases the protein content, which creates a higher amount of rennetable, gel-forming casein molecules, resulting in a more rigid structure.

Among the samples with ripened cheese, a common trend is observed, namely, increasing the amount of cheese results in lower values of hardness. The dispersed ripened cheese acts as an inert filler, therefore hindering the formation of a more continuous and homogeneous gel network, thus explaining the impact on hardness. However, as with the control samples without cheese, the addition of skimmed milk powder increased considerably the hardness of the gel, particularly in the presence of waxy rice starch.

The initial slope of the force-deformation curve gives an apparent modulus of elasticity of the gels (*44*). We evaluated such slopes for a representative number of samples (data not shown). While the control milk (CT) sample had a slope of 20 N/cm, in the controls with starch (CT.CS.1 and CT.WRS.1) the number decreased to 10-12 N/cm, that is, resulted in a softer gel. However, inclusion of skimmed milk powder (CT.CS.3 and CT.WRS.3) resulted in an almost 3-fold higher value (36-39 N/cm). Therefore, the inclusion of skimmed milk powder makes the gel significantly stiffer. Samples incorporating ripened cheese have lower elasticity than CT, regardless of having corn starch or waxy rice starch. These results are in alignment with those previously reported for hardness.

Regarding adhesiveness, control samples with starch, either corn or waxy rice starch, led to increased (negative) values. On the other hand, the addition of skimmed milk powder decreased adhesiveness of control samples. Samples incorporating ripened cheese showed lower adhesiveness than corresponding controls, and without a significant variation in the amount of cheese incorporation. We note that the relatively high values of standard deviation reported for adhesiveness in the same samples is a common feature of this analysis, particularly with heterogeneous matrices, as these ones (*45*, *46*). But one can conclude that starch is the ingredient with the main impact on this property.

As for the cohesiveness values, controls and samples with ripened cheese showed similar values among them, although the statistical analysis showed differences in a few cases. For instance, CT and CT.WRS.2, and CT and CT.WRS.1 have statistically significant differences (p<0.05), indicating that samples with 2 and 4% waxy rice starch are more cohesive than the basic milk control.

All samples showed springiness values close to 1, which indicates that they sprung back almost entirely after the first compression, albeit the above-mentioned gel breakage.

### Sensory properties of fresh cheese samples

A total number of 40 participants were included in the sensory analysis of the fresh cheese samples, predominantly young people, reflecting a university campus: 40% were below 24 years old, 37% between 25 and 34, 10% between 35 and 45, and 13% were above 45. The panel of participants reported a frequent consumption of dairy products, including cheese (83% of the participants), milk (75%) and yoghurt (65%), with a slightly higher preference for cheese, which makes them a good consumer panel for this product.

Regarding the different modes of consumption of fresh cheese, 63% of the consumers of the panel slice it over bread or toast, 55% consume it in salads, and 43% might also eat it by itself. The attributes most enjoyed in fresh cheese were reported to be freshness (75%), followed by texture (55%) and flavour (48%). Thirty percent of the participants are also interested in its low caloric content.

For the purpose of these sensorial tests, we selected the same ripened ewe’s cheese used in the above studies but substituted the (cow’s) Emmental for Cheddar cheese (CH), for its stronger flavour, and also included a goat’s cheese (GT). All three were used at 6.4% (*m*/*m*) in the fresh cheese samples. A fourth sample, made with Cheddar, included also skimmed milk powder for a firmer texture.

**Fig. 2:**
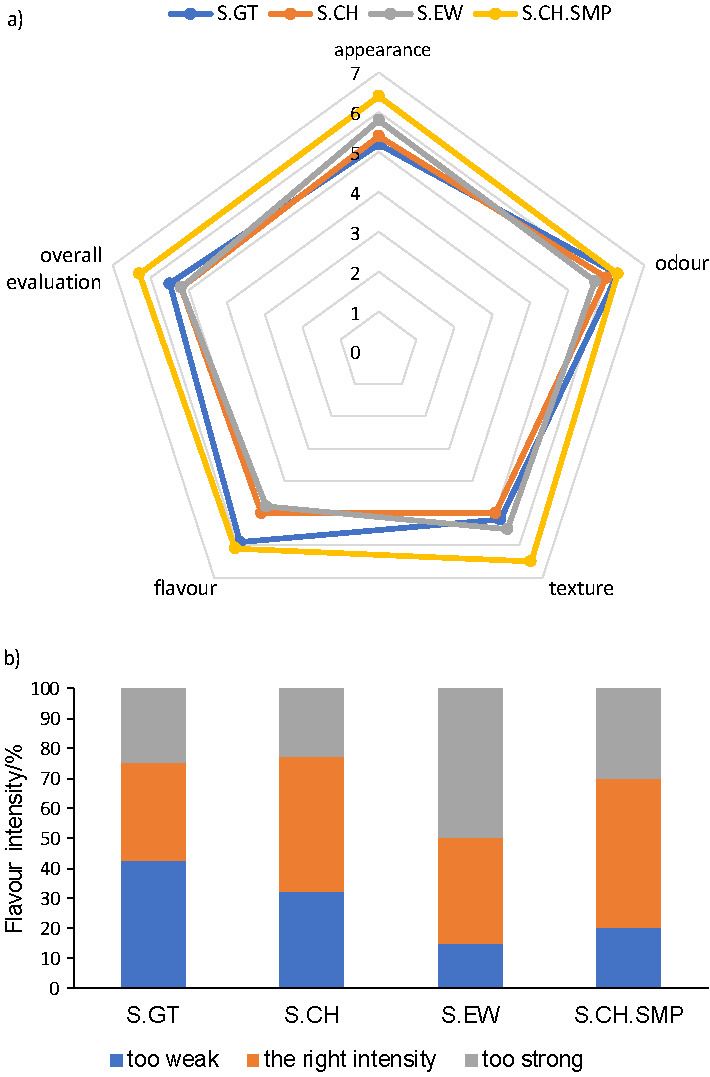
Panel evaluation of: a) different attributes and overall acceptability, and b) flavour intensity of different fresh cheese samples. S.GT, S.EW, S.CH are fresh cheese samples incorporating goat’s, ewe’s or Cheddar cheese; S.CH.SMP also incorporates skimmed milk powder (SMP)

The appearance, odour, texture, flavour and overall evaluation of fresh cheese were rated on a 9-point hedonic scale (where 1=dislike extremely, 2=dislike very much, 3=dislike moderately, 4=dislike slightly, 5=neither like nor dislike, 6=like slightly, 7=like moderately, 8=like very much and 9=like extremely). The participants showed preference for the sample with Cheddar cheese with added skimmed milk powder (S.CH.SMP), in all the attributes (Fig. 2a). This fresh cheese also received the highest overall evaluation, with an average of 6.3 points, followed by sample S.GT, with a rating of 5.5.

Regarding the intensity of flavour (Fig. 2b), 50% of the participants replied that sample S.CH.SMP was just right, and 45% had the same opinion on sample S.CH. Half of the panellists found the flavour of sample S.EWE too strong, and 42.5% found the flavour of sample S.GT weak. In fact, ewe’s cheese frequently has an intense flavour, to which many consumers are not familiarized, and/or not expect to find in a fresh cheese. The goat’s cheese used had a milder flavour intensity than either the Cheddar or the ewe’s cheese.

An additional comment made by 10 participants was that, regarding texture, the samples were more similar to yoghurt or quark cheese, rather than to a characteristic Portuguese fresh cheese, which has a denser texture. The relevance given to this property explains why the sample with highest rating was the one with skimmed milk powder (S.CH.SMP), which leads to a product with a considerably more consistent and cohesive texture. A recent work points as well to a correlation between the results of textural parameters and sensorial evaluation of acid-coagulated fresh cheese (*47*).

## CONCLUSIONS

Ripened cheese can be dispersed in hot gelatinized starch in milk and the resulting slurry (melted cheese base) can be an ingredient for a variety of food products. This represents a valorisation strategy for ripened cheese that could eventually become food waste. In this work, the melted cheese base was diluted further with fluid milk and then renneted, in order to obtain novel fresh cheese. That ingredient not only adds protein and fat to the fresh cheese, but it also adds minerals and flavour. A step of whey drainage is not included and the overall process is extremely simple. The developed fresh cheese has a valuable and balanced nutritional content, and a texture similar to many commercial fresh cheese types, or spreadable processed cheese. Furthermore, no emulsifying salts or any non-natural ingredients are used, enabling the classification of ’clean label’ and agreement with the sustainable development goals.

Gel formation of the initial mixture is hindered when too high amount of ripened cheese is added, but this can be overcome by the addition of skimmed milk powder (or rennetable casein) to the preparation. Starch and skimmed milk powder both reduce syneresis of the renneted gel. Starch seems to decrease gel hardness, but the addition of skimmed milk powder has a strong opposite effect. The sensory attributes of the product, such as texture and flavour, can be modulated by varying the amount and type of ripened cheese, and of extra casein (from skimmed milk powder or other). The consumer panel showed greater preference for a more solid texture and the flavour of a traditional cow’s cheese.

Therefore, the viability and versatility of this novel fresh cheese is here demonstrated. Further work can focus on minor adjustments of composition and modification of the production to the pilot scale level.

Fig. 1. Optical microscopy images, stained with: a and b) Rhodamine B, and c and d) Sudan III, of melted cheese bases prepared with: a and c) corn starch, and b and d) waxy rice starch
